# Effect of eco-friendly management of golf clubs on golfers’ behavioral intention to return: green image, perceived quality as mediator and green marketing as moderator

**DOI:** 10.3389/fpsyg.2025.1658880

**Published:** 2025-09-22

**Authors:** Kwon-Hyuk Jeong, Jeongmyeong Song

**Affiliations:** ^1^Department of Taekwondo, College of Physical Education, Kyung Hee University, Yongin-si, Gyeonggi-do, Republic of Korea; ^2^Department of Physical Education, College of Arts and Physical Education, Kangnam University, Yongin-si, Gyeonggi-do, Republic of Korea

**Keywords:** golf club, eco-friendly management, behavioral intention, green image, perceived quality, green marketing

## Abstract

**Introduction:**

Amid growing concerns about environmental sustainability, the sports and leisure industry has seen increased interest in green management practices. However, few empirical studies have explored how such practices influence consumer behavior in golf clubs. This study addresses that gap by examining how eco-friendly management affects golfers’ behavioral intention to revisit. Specifically, the study investigates the mediating roles of green image and perceived quality, and the moderating effect of green marketing in this relationship.

**Methods:**

To empirically test the proposed model, data were gathered from 252 South Korean golfers using structured field surveys. The questionnaire measured five constructs: eco-friendly management, green image, perceived service quality, green marketing exposure, and behavioral intention. Analytical methods included frequency analysis, confirmatory factor analysis (CFA) to test construct validity, reliability testing (Cronbach’s *α*), descriptive statistics, and Pearson’s correlation. To test mediation and moderation effects, regression-based analyses were conducted using Hayes’ (2013) PROCESS macro.

**Results:**

The statistical findings revealed three key insights. First, green image fully mediated the effect of eco-friendly management on behavioral intention, implying that consumers’ perception of the club’s environmental responsibility significantly influences their revisit decisions. Second, perceived quality also acted as a full mediator, indicating that environmental initiatives enhance service evaluations, thereby affecting loyalty behavior. Third, green marketing moderated the relationship between eco-friendly management and green image, suggesting that when marketing efforts are more active and visible, the positive impact on green image is amplified.

**Discussion:**

This research offers valuable contributions to the literature on environmental psychology and sustainable service management. It uncovers the psychological mechanisms—specifically green image and perceived quality—through which eco-friendly practices influence consumer behavior. Moreover, it highlights the role of green marketing in shaping environmental perceptions. For golf clubs aiming to enhance customer retention, this study suggests that sustainability initiatives must be effectively communicated and designed to improve both image and service quality. The results underscore the strategic importance of aligning operational greening with targeted environmental messaging.

## Introduction

The advancement of industrial technology in the 20th century significantly improved human life, but also led to serious environmental consequences, including pollution and climate change (Saxena, 2025). These environmental problems have evolved into complex global challenges that threaten human health, ecosystems, and long-term sustainability (Olorunsogo et al., 2024). In response, businesses have begun adopting eco-friendly strategies, and consumers increasingly prefer products and services that minimize environmental impact (Reddy et al., 2023). The sports industry has joined this shift as well, embracing eco-friendly initiatives to reduce its environmental footprint (Jang and Choi, 2025).

The sports industry also began implementing eco-friendly management. The US Major League Baseball (MLB) has pioneered the promotion of eco-friendly marketing activities since the early 2000s. MLB has been setting an eco-friendly trend within the sports industry, such as introducing the Leadership in Energy and Environmental Design (LEED)-certified buildings and encouraging active Eco-friendly participation at the team level. The Korean Professional Football League launched the “Green Kick-off” campaign in 2021, aiming to reduce carbon and greenhouse gas emissions in stadiums. It initiates the movement to solve environmental issues with gamification strategies, such as developing games or video content that engages fans. SK Wyverns, a former professional baseball team in South Korea, had gained attention with their “Green Sports” initiative to implement green marketing strategies (Lee, 2010). This team introduced renewable energy facilities in its home stadium in cooperation with local governments and public institutions and developed products that use eco-friendly materials, presenting a sustainable development model in the professional sports industry. The SK Wyverns case significantly contributed to advancing eco-friendly marketing in South Korean professional sports and continues to be recognized by both academia and the industry as a notable example. Likewise, many European sports organizations have recently pledged to achieve net-zero emissions and integrate sustainability into operations (Khanna et al., 2024), reflecting a worldwide trend of greener practices in sports.

Golf is a sport that requires vast areas of land, and the construction and operation of golf clubs may lead to environmental challenges in land use, disruption of ecosystems, and depletion of water resources (Fouillouze et al., 2023). In particular, pesticides and fertilizers used in golf club maintenance can pollute soil and water, causing severe damage to ecosystems and negatively impacting the surrounding environment (Sorrentini, 2022). In their maintenance, large amounts of water are consumed, and chemical substances are used excessively, reducing soil fertility and causing ecosystem imbalance.

The Korea Golf Course Business Association (KGBA) recognized the severity of these environmental issues and adopted environmental, social, and governance (ESG) management when pursuing sustainable growth, to promote the sustainable development of the golf industry. Such a management approach ensures a company’s long-term growth and stability while contributing to the creation of social value (Kim and Li, 2021). According to a survey by Yeom et al. (2023), 73.2% of South Koreans were interested in environmental issues. According to the “Survey on Public Perception of ESG Management and Companies” conducted by The Korea Chamber of Commerce & Industry (2021), more than 60% of all consumers consider companies’ ESG activities when purchasing their products. In response, KGBA has introduced reclaimed water use, environmental audits, and native species planting as key ESG practices (KGBA Annual Report, 2023). Internationally, clubs like Sentosa Golf Club in Singapore have achieved carbon neutrality and implemented fully electric cart fleets (Sentosa Golf Club, 2023). These findings indicate a growing societal awareness of ESG factors and an increasing demand for sustainable practices from businesses.

Previous research on green marketing focused primarily on understanding why consumers purchase eco-friendly products (Kaufmann et al., 2012) and on consumer loyalty based on eco-friendly experiences (Wu et al., 2018). In eco-friendly studies on golf clubs, studies have been conducted on the ecosystem services of golf clubs for urban planning (Rice and Horgan, 2017) and the eco-friendly sustainability of regional golf clubs (Keogh et al., 2014). More recently, researchers have also begun investigating sustainability in sports services and tourism, reflecting broader global interest in green consumer behavior (e.g., Li et al., 2023; McCullough, 2023;).

However, despite increasing public interest in sustainable consumption, few studies have empirically examined how eco-friendly golf course management affects specific consumer behavioral constructs—such as revisit intention, perceived quality, and attitudinal loyalty—which are critical to both theoretical development and managerial practice in service-oriented leisure sectors (Costa et al., 2024; Morán-Gámez et al., 2024). This research addresses a notable gap in the literature, as these constructs—though grounded in the Natural-Resource-Based View (NRBV) and Service Quality Theory—remain underexplored in the context of ESG-driven golf marketing.

In light of this research gap, the necessity of the current study becomes particularly evident. While several studies have addressed sustainable behaviors in general sports and leisure contexts—such as spectators’ eco-conscious choices in professional sports venues (McCullough, 2023), tourists’ green satisfaction in eco-resorts (Li et al., 2023), and the impact of green branding on sport clubs users’ retention (Morán-Gámez et al., 2024)—few have focused specifically on the golf industry, despite its direct and ongoing interaction with ecological environments. Given golf’s dependency on land, water, and energy, the sport represents a high-impact domain for evaluating eco-friendly strategies.

Moreover, unlike general tourism or stadium-centered studies, golf clubs operate as both consumption and maintenance entities—where players experience and simultaneously assess environmental practices during use. This dual exposure reinforces the importance of perceived quality and green image as mediators of behavioral intention, especially in repeat-use leisure contexts like golf (Kim and Jeong, 2024). Accordingly, this study contributes uniquely by combining environmental, psychological, and marketing perspectives within the operational ecosystem of golf services (Cifci et al., 2025).

Eco-friendly practices, often synonymous with “green” or “environmentally responsible” behaviors, involve delivering products and services that minimize natural resource consumption and reduce environmental harm (Chen et al., 2015). As environmental, social, and governance (ESG) practices become increasingly central to corporate strategy, the golf industry has begun actively implementing ESG-driven approaches to promote environmental sustainability. This trend is particularly salient in golf, where the sport inherently interacts with and impacts ecological systems. Thus, golf clubs are increasingly adopting sustainability-driven management models to align with broader ESG objectives and generate long-term environmental and social value.

Yet beyond ecological stewardship, a fundamental question arises: “How does environmental management tangibly influence golfers’ behavioral intentions in a discretionary leisure context such as golf?” Addressing this question necessitates drawing upon integrative frameworks like the Natural-Resource-Based View (NRBV) (Hart, 1995), both of which posit that environmentally responsible practices can shape consumer attitudes, enhance perceived service value, and ultimately drive revisit intention (Liu et al., 2025). By adopting ESG elements in sustainable management models, golf clubs that are directly exposed to the environment can contribute to the development of local communities and mitigate the negative impact they have on the environment (Sorrentini, 2022). This can make golf clubs achieve sustainable development and gain the potential to positively influence local communities and the environment (Dal Mas et al., 2022). Such efforts pave the way for a brighter future while ensuring that golf clubs adhere to ethical standards.

Therefore, the present study seeks to empirically examine the indirect effects of eco-friendly management on behavioral intention via green image and perceived quality, while also testing the moderating effect of green marketing within this relationship. This study aimed to analyze the impact of eco-friendly management practices at golf clubs on golfers’ intentions to return to the respective golf clubs (hereafter referred to as ‘behavioral intention’ for conciseness) through mediating variables of green image and perceived quality. It also examines the moderating effect of green marketing on this relationship. Ultimately, the study aimed to propose effective green marketing strategies that encourage environmentally friendly consumer behaviors among golfers. Further, it sought to provide an academic foundation for the sustainable development of the golf industry and offer practical, eco-friendly management solutions for golf club operators.

## Literature review

### Eco-friendly and ESG management in golf course operations: a resource-based perspective

This study addresses this gap by positioning ESG practices as strategic drivers of consumer engagement and pro-environmental behavior in leisure sport contexts, particularly within golf course operations.

From the perspective of the Natural-Resource-Based View (NRBV) of the firm (Hart, 1995), ESG-aligned practices are not merely operational choices but constitute key strategic resources that can create sustainable competitive advantage. According to NRBV, firms that develop capabilities for pollution prevention, product stewardship, and sustainable development can simultaneously achieve environmental and economic performance (Alkaraan et al., 2024). In the case of golf course operations, environmental capabilities such as efficient water management, ecosystem restoration, and biodiversity conservation enhance the organization’s legitimacy and public trust (Lee and Kwon, 2024)—critical assets in service-based leisure industries. These findings align with the recent empirical work by Han and Niu (2023), who applied NRBV in a sports sustainability context and confirmed that eco-friendly facility practices significantly influence revisit and recommendation intentions.

Eco-friendly management, situated within the broader paradigm of Environmental, Social, and Governance (ESG) practices, reflects an organization’s strategic approach to reducing its environmental impact while fostering social inclusion and transparent governance (Mishra, 2023). In the context of golf, this translates into operational practices such as pesticide-free turf maintenance, integrated pest management, installation of renewable energy facilities (e.g., solar panels for clubhouse power), and use of reclaimed or rainwater for irrigation. ESG management in the golf industry encompasses three primary domains: environmentally responsible operations (e.g., organic turf care, renewable energy use), inclusive service access (e.g., community equity in course participation), and institutional transparency (e.g., third-party certifications such as GEO Certified and ISO 14001) (Goodland and Daly, 1996; Maloni and Benton, 1997; Korea Sustainability Investing Forum, 2022).

Given the growing visibility of ESG-oriented operations in the golf industry, it is essential to understand how such practices are perceived by consumers and how they shape behavioral outcomes. This study adopts a Resource-Based Perspective and the ESG management framework to empirically investigate how eco-friendly practices implemented by golf courses elicit consumer responses, such as revisit intention, recommendation, and pro-environmental behavior.

### Neo-environmental paradigm and mediating role of green image

The Neo-Environmental Paradigm (NEP) offers a conceptual lens through which consumers’ pro-environmental worldviews are understood (Dunlap and Van Liere, 1978). From this perspective, green image functions as an interpretive filter shaped by eco-management visibility, linking corporate environmental actions to cognition and emotion—for example, via consumer trust and emotional alignment. When golf courses engage in visible sustainability initiatives—such as attaining GEO Certification or publishing environmental performance reports—they reinforce trust and emotional alignment with ecological values (Chen, 2010).

Recent research in leisure and tourism supports this mechanism: in hospitality, Huyen et al. (2025) found green image enhances trust and goodwill, indirectly increasing revisit intention, while Lin et al. (2025) demonstrated that green image mediates relationships between sustainable service provision and customer loyalty in eco-resorts. Building on this framework, the current study posits that green image operates as a mediator—rather than a direct predictor—translating eco-management practices into behavioral outcomes such as return visits and brand advocacy, through a cognitive-affective pathway (Babiak and Trendafilova, 2011).

### Service quality theory and mediating role of perceived quality

In the service sector, service quality is often understood as the outcome of objective or process-based assessments, such as reliability, responsiveness, and professionalism (Grönroos, 1984; Parasuraman et al., 1988), whereas perceived quality refers specifically to the subjective evaluation formed by consumers based on their personal experiences and expectations. In the context of golf, eco-friendly practices—such as pesticide-free turf maintenance and solar-powered facilities—not only enhance the experiential value but also signal high service standards. These practices foster consumer trust, reinforce perceptions of professionalism, and ultimately drive satisfaction, loyalty, and behavioral commitment. In sports settings, where hedonic and experiential dimensions are central, environmental attributes have become increasingly influential in shaping perceived service quality (Lee, 2022).

Recent empirical studies support the mediating role of perceived service quality in sustainability contexts. For example, Zareh et al. (2023) found that eco-friendly initiatives in leisure resorts enhanced tourists’ perceived service quality, which in turn led to stronger revisit intention and satisfaction. Similarly, Kim (2021) demonstrated that sustainable facility management positively shaped perceived quality and behavioral responses among sports venue users.

Building on these insights, this study proposes that perceived quality functions as a mediating mechanism linking eco-friendly management to golfers’ behavioral intentions, by strengthening consumers’ evaluations of service excellence.

### Green marketing as a moderator of ESG and behavioral intentions

Green marketing facilitates consumer awareness of sustainability initiatives and shapes perceptions of brand authenticity (Huang and Guo, 2021; Rahman and Nguyen-Viet, 2023; Rana et al., 2025; Sujanska and Nadanyiova, 2024). It serves as a moderator that can either strengthen or weaken the impact of ESG practices on consumer intentions. As such, when green marketing is perceived as authentic and aligned with consumer values, its enhanced visibility and credibility amplify the effects of eco-friendly management by increasing the salience of these practices in consumers’ minds—therefore functioning as a moderator rather than a mediator (Kumar et al., 2021; Tan et al., 2025). When green marketing is perceived as authentic and consistent with consumer values, it heightens brand credibility, facilitates recognition of ESG efforts, and ultimately magnifies behavioral responses such as loyalty or purchase preference (Li and Kim, 2025; Ünal et al., 2024).

Recent studies support this moderating role across industries—for example, in the hospitality industry, Lee et al. (2019) found that green promotional practices strengthened the link between eco-certifications and guest satisfaction, and in retail, Kumar and Basu (2023) showed that eco-label visibility moderated the relationship between sustainable pricing and consumer purchase intention. Accordingly, this study hypothesizes that green marketing positively moderates the relationship between eco-friendly management and behavioral intentions by enhancing the perceived relevance and trustworthiness of corporate sustainability efforts.

## Theoretical basis and research hypothesis

### The impact of eco-friendly management on behavioral intentions

Corporate Social Responsibility (CSR) has long been recognized as a key element of corporate sustainability. Sustainability, according to Goodland and Daly (1996), encompasses economic, social, and environmental dimensions, with the environmental aspect increasingly gaining traction due to global regulations and public demand (Usman et al., 2024). In the context of golf services, eco-friendly management manifests as visible efforts aligned with ESG values, directly influencing how consumers form behavioral intentions (Myung, 2024).

In the context of golf services, eco-friendly management—such as pesticide-free turf care, water reuse systems, and renewable energy integration—may positively shape consumers’ behavioral intentions by influencing individual psychological processes as described in the Natural-Resource-Based View (Hart, 1995). Specifically, these visible environmental practices may foster favorable attitudes (by increasing moral satisfaction), strengthen subjective norms (through social endorsement of green behavior), and enhance perceived behavioral control (by providing accessible green alternatives), thereby increasing the likelihood of revisits, recommendations, and loyalty (Terason et al., 2022; Wang and Butkouskaya, 2023).

While classical literature often addresses CSR’s impact on corporate performance (Maloni and Benton, 1997; Zhu and Sarkis, 2004), recent studies have begun to explore how environmentally responsible practices influence consumer behaviors. In the context of tourism and hospitality, research indicates that such practices foster positive attitudes and behavioral intentions, such as revisit likelihood and recommendation (Han et al., 2010; Li et al., 2022). For instance, Zarei et al. (2024) found that customers’ perceptions of eco-friendly practices in leisure settings positively influenced their pro-environmental behavioral intentions through emotional and normative engagement.

These insights are highly relevant to the golf sector, where natural surroundings and sustainability are core service attributes. Given that golf courses often operate in ecologically sensitive environments, visible efforts toward sustainability may enhance customer satisfaction, loyalty, and willingness to revisit, making eco-friendly management a vital factor in shaping golfer behavior.

*H1:* Eco-friendly management at golf courses will have a positive impact on golfers' behavioral intentions.

### Mediating effect of green image on the relationship between eco-friendly management and golfers’ behavioral intentions

Green image is defined as the extent to which consumers perceive an organization as environmentally responsible (Alam and Islam, 2021). According to Moffitt (1994), organizational image results from intentional positioning and public perception. Sharma and Bansal (2013) found that visible commitment to environmental responsibility enhances organizational appeal and trust. In this context, green image acts as a cognitive filter that enables consumers to interpret eco-friendly management as aligned with their personal values.

Joo and Lee (2024) demonstrated that ESG activities in sports contexts improve green image and customer retention. Particularly in the golf industry, where consumers often associate course aesthetics and maintenance with brand image, eco-friendly practices—such as chemical-free turf and energy-efficient facilities—could strengthen green image and indirectly increase behavioral intention. Recent international studies further support this mediating mechanism. For example, García et al. (2017) found that green image significantly mediated the relationship between sustainable practices and customer loyalty in the hospitality sector, emphasizing its role in translating environmental values into behavioral outcomes.

Similarly, Setiyarini et al. (2022) demonstrated in a tourism context that consumers’ perception of eco-friendly branding enhanced brand image, which in turn increased revisit intentions and emotional brand attachment. These findings suggest that in service environments where environmental cues are salient—such as golf courses—green image can serve as a powerful mediator that transforms operational sustainability into attitudinal and behavioral commitment.

*H2:* The green image of a golf club will mediate the relationship between eco-friendly management and behavioral intentions.

### Mediating effect of perceived quality on the relationship between eco-friendly management and golfers’ behavioral intentions

Perceived quality refers to the customer’s judgment of the overall excellence of a product or service. Perceived quality is described as the result of comparing customer expectations with perceived performance (Grönroos, 1984). This concept is particularly salient in service-oriented sectors like golf, where consumer evaluations are shaped by both tangible (e.g., course conditions) and intangible (e.g., environmental values) elements.

In the sports industry, recent research supports the idea that eco-friendly management enhances perceived service quality by improving environmental integrity, facility hygiene, and service professionalism. For example, Cuesta-Valiño et al. (2021) found that sustainability initiatives in sports facilities positively influenced users’ evaluations of quality and safety, leading to greater satisfaction and loyalty. Similarly, Liu et al. (2024) demonstrated that spectators’ perception of environmental responsibility in events significantly enhanced their perceived event quality, which in turn increased their intention to revisit.

Eco-friendly management may enhance perceptions of cleanliness, professionalism, and credibility. These improved perceptions of quality can, in turn, reinforce golfers’ intentions to revisit or recommend the venue.

*H3:* Perceived quality will mediate the relationship between the golf club’s eco-friendly management and golfers’ behavioral intentions.

### Moderating effect of green marketing on the relationship between eco-friendly management and golfers’ behavioral intentions

Green marketing refers to promotional efforts that highlight a company’s commitment to sustainability through product development, communication, and distribution strategies (Peattie and Crane, 2005). Effective green marketing informs consumers about the company’s environmental initiatives, enhancing the perceived value of those efforts and fostering deeper behavioral engagement (Kotler and Armstrong, 2003).

Critically, green marketing may act as a moderator by amplifying or attenuating the effect of eco-friendly management on behavioral intention. When a golf club actively communicates its green practices—through promotional campaigns, eco-certifications, or signage—customers are more likely to perceive those actions as credible and compelling (Jain and Kaur, 2004; Papadopoulos et al., 2010). Without such visibility, the behavioral impact of eco-friendly management may be diluted.

Recent international research supports this moderating role. For instance, Nosrati et al. (2024) demonstrated that eco-label visibility enhanced the relationship between environmental practices and customer engagement in green hospitality settings. These findings suggest that strategic green marketing communication can heighten the effectiveness of eco-friendly management in the context of recreational services such as golf.

*H4:* Green marketing will moderate the relationship between the golf club’s eco-friendly management and golfers’ behavioral intentions.

**Figure 1:**
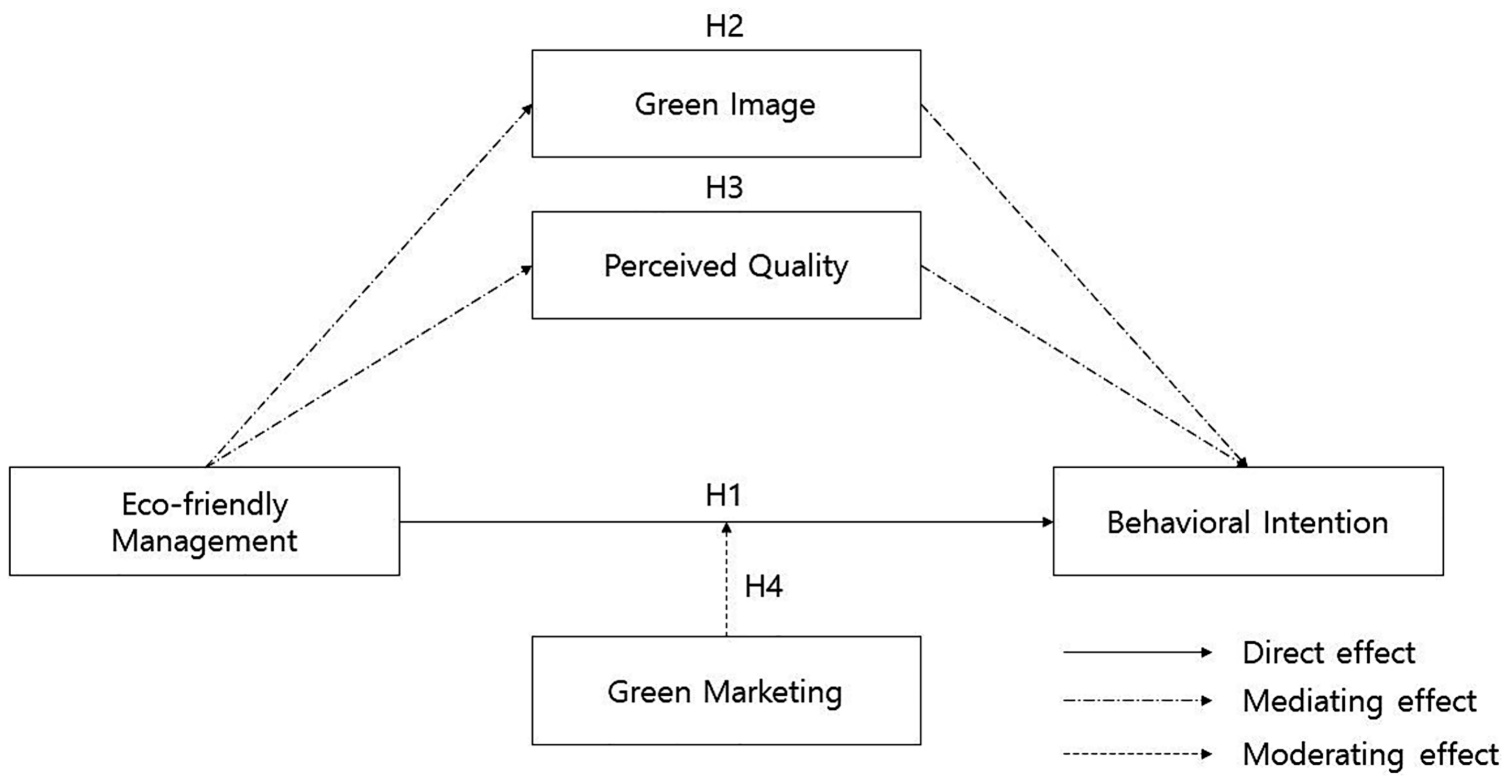
Research model.

Accordingly, the conceptual research model proposed in this study is presented in Figure 1.

## Methods

### Ethics approval and consent to participate

This study was approved by Public Institutional Review Board designated by Ministry of Health and Welfare (No. P01-202409-01-007) on January 9, 2025. All methods performed in studies involving human participants were in accordance with the ethical standards of the Institutional Review Board of Public Institutional Review Board designated by Ministry of Health and Welfare and with the 1964 Helsinki declaration and its later amendments or comparable ethical standards. Informed consent was obtained from all individual participants included in the study during the period of January 10 to February 20, 2025.

### Participants

The participants of this study were South Korean nationals aged 19 and older who had visited a golf club and engaged in golf activities in the past year. A convenience sampling method, a type of non-probability sampling, was employed. The survey was conducted for 2 months, by directly visiting six locations that had responded to an initial survey to determine which local facilities would be willing to participate: two golf clubs, three golf practice ranges, and one screen-golf facility in the metropolitan area of South Korea. Information sheets explaining the study were displayed, and individuals interested in participating were recruited. The survey was administered only to those who consented after we had explained the study to them. These self-administered surveys were collected immediately upon completion. Surveys with incomplete or poor-quality responses were excluded from the analysis.

Although the survey was conducted entirely in South Korea and the items were administered in Korean, we implemented a rigorous cultural adaptation process to enhance the measurement validity. All original measurement instruments were developed in English, and the Korean version was professionally translated by a bilingual expert with domain-specific knowledge. A separate bilingual translator, blinded to the original, independently back-translated the items into English. The research team reviewed and reconciled any discrepancies between the source and back-translated versions to ensure conceptual equivalence. Furthermore, the Korean items were examined for cultural relevance, including locally meaningful expressions, behavioral patterns, and perceptions of eco-friendly practices in the context of South Korean golf consumption.

While this study was geographically limited to South Korea, we identified several cultural and behavioral characteristics—such as heightened consumer sensitivity to ESG branding and eco-certified services—that are increasingly common in other developed markets. Therefore, the insights drawn from this research may offer implications for eco-friendly management strategies in global golf settings as well.

In this study, the sample size required for the analysis of mediating and moderating effects was assessed to ensure statistical adequacy. Following the method outlined by Lee (2016), the G*power 3.1.9.7 program was utilized to calculate the required sample size with an effect size of 0.15, a significance level of 0.05, a power of 0.95, and a total of 8 predictors (including control variables, independent variables, moderating variables, and interaction terms). The results indicated that the minimum required sample size was 160 participants. However, considering a 30% attrition rate, it was determined that a final sample size of at least 208 participants would be necessary. Therefore, the study collected a total of 260 completed surveys.

A total of eight such surveys were removed, resulting in a final sample size of 252 respondents. Table 1 presents the characteristics of the participants.

**Table 1:** Participant characteristics.

Variable	Category	*N*	Percentage (%)
Gender	Male	179	71.0
	Female	73	29.0
Age	20s	52	20.6
	30s	99	39.3
	40s	43	17.1
	over 50s	58	25.0
Educational background	High school graduate	31	12.3
	Bachelor’s	90	35.7
	Master’s	131	52.0
Experience	Less than 10 years	145	57.5
	More than 10 years	107	42.5
Number of visits	Once a month	135	53.6
	Twice a month	55	21.8
	Once a week	31	12.3
	More than twice a week	31	12.3
Total		252	100

### Measurement

Before conducting the survey, examples of eco-friendly management practices at golf clubs were presented to the participants, as shown in Table 2.

**Table 2:** Examples of eco-friendly management of golf clubs.

Golf club that makes efforts and implements actions to reduce carbon emissions (lowering greenhouse gas emissions) Golf club that minimizes the use of chemical fertilizers and pesticides (microbial pesticide, alternative organic pesticide) Golf club that minimizes soil cutting volume Golf club that utilizes eco-friendly materials (LEDs, wood, stone, natural grass, etc.) in its club house, shades, and courses Golf club that preserves significant areas of virgin forests Did ecological and green experts participate from the stage of site selection? Did the designer design the course to preserve original green areas before assigning play functions? Golf club where areas with healthy forest clusters or streams capable of supporting aquatic ecosystems were preserved as much as possible Golf club that installed natural streams or waterfalls when designing water features to ensure water movement Golf club that incorporated solar power or geothermal energy into club house designs Golf club that used permeable materials for golf cart paths and eco-friendly grass blocks for parking areas

Measured variables comprised eco-friendly management practices, behavioral intentions, golf club image, and green marketing. All items were structured on a 7-point Likert scale. Additionally, golfers’ demographic characteristics were assessed by gender, age, educational background, golfing experience, and number of visits.

The Eco-friendly Management Scale are evaluations of the environmental protection activities of golf clubs, efforts to establish protection plans and practice environmental protection, and operation of dedicated departments for environmental protection and strategies for environmental management involving investments and actions (Korea Corporate Governance Service, 2024; Lee, 2022; Park and Han, 2021). This was structured as a single-factor construct comprising six items. For this study, higher scores on the 7-point Likert scale indicate greater efforts by the golf club in their eco-friendly management.

The Behavioral Intentions Scale measures to determine the participants’ willingness to revisit a golf club that engages in eco-friendly management. Kerstetter et al. (2004), Thapa (2010), and Chiu et al. (2014) set a framework within the eco-friendly consumption section of behavioral intention that aligns with behaviors after visiting golf clubs. This was structured as a single-factor construct comprising four items. In this study, higher scores on the 7-point Likert scale indicate a greater willingness to revisit the golf club with eco-friendly management.

The Green Image Scale refers to the eco-friendly image of the golf clubs. It was refined with reference to the measurement scale used by Hou (2024) who confirmed the corporate image, and in line with the golf image used by Lee and Kang (2016), and Panthasupkul and Phromphithakkul (2021). This was structured as a single-factor construct comprising seven items. In this study, higher scores on the 7-point Likert scale indicate a more positive image of golf clubs.

The Perceived Quality Scale measures the level of satisfaction that golfers feel regarding the quality of their experiences at a golf club. We adapted the scale used in Lee and Hwang (2014), based on the studies of Chen and Chen (2010) and Otto and Ritchie (1996), to best fit golfers. This was structured as a single-factor construct comprising four items. In this study, higher scores on the 7-point Likert scale indicate a higher quality of golf clubs.

The Green Marketing Scale refers to the golfers’ direct experiences of green marketing by golf clubs. Green marketing variables from studies by Reddy et al. (2023), and Javed et al. (2024) were adapted to fit the context of golf clubs. This was structured as a single-factor construct comprising three items. In this study, higher scores on the 7-point Likert scale indicate a more positive response to green marketing by golf clubs.

### Data analysis

Data were analyzed using IBM SPSS Statistics 27.0. First, frequency analysis was conducted to identify the demographic characteristics of the respondents. Second, convergent validity and discriminant validity were analyzed with confirmatory factor analysis (CFA) through unidimensionality verification to check the validity of the survey. Third, to assess the reliability of each factor, internal consistency was tested using Cronbach’s *α*. Fourth, following the suggestion by Dabholkar et al. (1996), mediation and moderation analyses were conducted using HAYES’ macro model to apply a partial distribution model, which divides the total sum of the constructs of each variable used in the study by their averages.

## Results

### Scale validity and reliability

To verify the validity of the scale that measured multiple items, this study conducted confirmatory factor analysis (CFA). The input data for the model testing was based on the correlation matrix. The convergent validity and discriminant validity were verified before verifying the overall fit of the research model.

#### Verification of convergent validity and reliability

The measurement model for the overall research was analyzed, and the results are presented in Table 3. CFA results indicated a good model fit, with X^2^ = 418.469 (*df* = 242, *p* < 0.001), *X^2^/df* = 1.729, normed fit index (NFI) = 0.901, Tucker-Lewis Index (TLI) = 0.949, comparative fit index (CFI) = 0.955, and root mean square error of approximation (RMSEA) = 0.054, which verifies convergent validity (Browne and Cudeck, 1993). The analysis of latent variables revealed that convergent validity is supported if the standardized factor loadings of observed variables on their respective latent variables were statistically significant (*p* < 0.05).

**Table 3:** CFA results for all variables.

Question	*β*	S.E.	*t*
Eco-friendly management (EM)			
Optimistic about golf clubs planning and implementing eco-friendly management.	0.739	–	–
Optimistic about golf clubs working to implement environmental protection plans.	0.755	0.086	11.508
Optimistic about golf clubs operating a dedicated division for environmental protection.	0.732	0.088	11.140
Optimistic about golf clubs effectively conducting evaluations on outcomes for environmental management.	0.696	0.085	10.581
Optimistic about golf clubs investing well in facilities and equipment within the golf club.	0.719	0.085	10.946
Optimistic about golf clubs continuously working toward environmental protection.	0.743	0.085	11.310
Policy consistency (AVE = 0.534, C. R. = 0.873, Cronbach’s α = 0.873)			
Green image (GI)			
If golf clubs practice eco-friendly management, its reputation will enhance.	0.703	–	–
Eco-friendly golf clubs are likely to have high-quality facilities.	0.753	0.101	11.294
Eco-friendly golf clubs are likely to provide differentiated services.	0.835	0.098	12.457
Eco-friendly golf clubs are likely to have a luxurious image.	0.816	0.097	12.192
Eco-friendly golf clubs are likely to provide reliable services.	0.822	0.092	12.280
Eco-friendly golf clubs are likely to have a good impression.	0.785	0.086	11.743
Eco-friendly golf clubs are likely to have been beautifully landscaped.	0.768	0.095	11.508
Policy consistency (AVE = 0.615, C.R. = 0.918, Cronbach’s α = 0.917)			
Perceived quality (PQ)			
Eco-friendly golf clubs are likely to give me a great experience that I will remember.	0.862	–	–
Golfing at an eco-friendly golf club is likely to be interesting and new.	0.867	0.063	17.654
Golfing at an eco-friendly golf club is an experience that should be had at least once in a lifetime.	0.819	0.067	16.104
Golfing at an eco-friendly golf club is likely to be an opportunity for relaxation.	0.848	0.062	10.581
Policy consistency (AVE = 0.721, C.R. = 0.912, Cronbach’s α = 0.911)			
Green marketing (GM)			
Eco-friendly golf clubs seem like they will offer eco-friendly services through shared value (green and clean) events.	0.903	–	–
Eco-friendly golf clubs seem like they will put in effort to reduce energy consumption and reduce greenhouse gases.	0.937	0.046	25.394
Eco-friendly golf clubs seem like they have established appropriate eco-friendly plans and directions.	0.967	0.041	27.559
Policy consistency (AVE = 0.876, C. R. = 0.955, Cronbach’s α = 0.954)			
Behavioral intention (BI)			
I am interested in utilizing an eco-friendly golf club.	0.748	–	–
I am willing to visit an eco-friendly golf club.	0.842	0.079	11.446
I want to visit an eco-friendly golf club.	0.675	0.085	9.841
I feel positively about visiting an eco-friendly golf club.	0.648	0.078	9.457
Policy consistency (AVE = 0.536, CR = 0.821, Cronbach’s α = 0.813)			

Model fit	X^2^	*df*	*p*	Q	NFI	TLI	CFI	RMSEA
	418.469	242	***	1.729	0.901	0.949	0.955	0.054

β = standardized coefficient; S.E. = standard error; *t* = *t*-value; p = probability value. Significance levels: ***p < 0.001.

As shown in Table 3, all items had statistically significant *t*-values. Cronbach’s α values for the constructs ranged from.813 to.954, demonstrating high reliability. The Construct Reliability (C.R.) values, calculated using the estimated factorial load values, were found to be between 0.821 and 0.955, and the average variance extracted (AVE) values between.534 and.876. These values meet the acceptable criteria (C.R. ≥ 0.7, AVE ≥ 0.5), indicating that reliability has been established (Fornell and Larcker, 1981).

#### Verifying discriminant validity and correlation

To establish discriminant validity, there must be a difference in the measurement results of different constructs. This study applied the verification method proposed by Anderson and Gerbing (1988) and Fornell and Larcker (1981). Using the correlation matrix (*φ* matrix), the values calculated within a 95% confidence interval [correlation ± (2 × standard error)] should not include 1. If the AVE value is larger than the squared correlation among constructs, discriminant validity is established. The squared correlation values among all variables ranged from 0.001 to 0.247, and the AVE value (latent factor) ranged from.534 to.876, establishing partial discriminant validity. As shown in Table 4, the examination of all variables revealed significant differences between them. Therefore, discriminant validity was established through the above verification method.

**Table 4:** Verifying discriminant validity and correlation.

Category	1	2	3	4	5
Environment	0.534*^(1)^	0.117^(3)^	0.116	0.156	0.001
Image	0.342**^(2)^	0.615	0.227	0.202	0.036
Quality	0.340**	0.476**	0.721	0.247	0.040
Green	0.578**	0.449**	0.497**	0.876	0.026
Behavior	0.083	0.189**	0.199**	0.160*	0.536
Mean	5.213	5.604	5.794	5.340	6.262
S.D.	1.213	0.947	1.082	1.457	0.753
Skewness	−0.725	−0.836	−0.769	−0.663	−1.039
Kurtosis	−0.173	0.664	−0.064	−0.313	0.449

^(1)^AVE (Average Variance Extracted), ^(2)^*r*, ^(3)^*r*^2^. Significance levels: *p < 0.05, **p < 0.01.

### Verifying the hypotheses

To test Hypothesis 1, a simple linear regression analysis was conducted by using the SPSS program. To verify H2 and H3, the mediating effect was analyzed with PROCESS macro model 4 by Hayes (2013). The bootstrapping method with 5,000 iterations was used to verify the mediating effect. In addition, to verify H4, the moderating effect was analyzed with PROCESS macro model 1. Following the suggestions of Aiken and West (1991), conditional effects were examined to explore the moderating effects in detail.

#### The impact of eco-friendly management on behavioral intentions

The analysis results for Hypothesis 1, as presented in Table 5, revealed that the relationship was not statistically significant (*F* = 1.720, *p* > 0.05). Furthermore, the explained variance was found to be very low, at only 3% (*R*^2^ = 0.003).

**Table 5:** The direct impact of eco-friendly management on behavioral intention.

Category	B	SE	*β*	*t*	*p*
Eco-friendly management	0.051	0.039	0.083	1.312	n.s.
*F* = 1.720, Adjusted *R*^2^ = 0.003, *p* > 0.05					

B = unstandardized coefficient; β = standardized coefficient; S.E. = standard error; *t* = t-value; p = probability value. Significance levels: n.s. = non-significant.

This finding suggests that eco-friendly management, in isolation, may not directly trigger behavioral intentions among golf consumers. One possible explanation is that consumers often require additional cognitive or emotional mechanisms—such as trust, brand image, or service quality perceptions—to translate observed sustainability efforts into concrete behavioral outcomes. This aligns with previous research highlighting that the effects of CSR and ESG practices are often indirect, operating through mediators such as perceived value or moral alignment rather than exerting direct influence. It is also possible that green efforts are not yet salient or personally relevant enough for all customers to motivate action unless reinforced through communication or experiential engagement.

#### Mediating effect of green image

As shown in Table 6, the analysis of the mediating effect of green image indicates that eco-friendly management practices in golf clubs did not have a direct effect on golfers’ behavioral intention (*β* = 0.072, *t* = 1.802, *p* > 0.073). If the 95% bootstrap confidence interval does not include zero, the indirect effect is considered statistically significant, indicating mediation. This study found a complete mediating effect (*β* = 0.057, [CI = 0.017–0.106]). Complete mediation was supported, as the direct effect of eco-friendly management became non-significant when the indirect path via green image was statistically significant.

**Table 6:** Analysis results of the mediating effect of green image.

Category	*β*	SE	*t*	*p*	LLCI	ULCI
Total effect	**0.108**	**0.039**	**2.787**	**	**0.032**	**0.184**
Direct effect	0.072	0.040	1.802	n.s.	−0.007	0.152
Indirect effect (Standard)	**0.035(0.057)**	**0.014(0.023)**	**-**	**-**	**0.010(0.017)**	**0.066(0.106)**

β = standardized coefficient; S.E. = standard error; *t* = t-value; p = probability value; LLCI = lower-level confidence interval; ULCI = upper-level confidence interval. Significance levels: **p < 0.01; n.s. = non-significant. Bold values indicate statistically significant results at *p* < 0.05.

#### Mediating effect of perceived quality

As shown in Table 7, the analysis of the mediating effect of perceived quality indicates that eco-friendly management practices in golf clubs did not have a direct effect on golfers’ behavioral intention (*β* = 0.066, *t* = 1.645, *p* > 0.101). Following the bootstrap-based mediation approach, the indirect effect was confirmed when the confidence interval did not contain zero (*β* = 0.067, [CI = 0.015–0.125]). A statistically significant effect was observed in the indirect path, indicating the presence of an indirect effect.

**Table 7:** Analysis results of the mediating effect of perceived quality.

Category	*β*	S.E.	*t*	*p*	LLCI	ULCI
Total effect	**0.108**	**0.039**	**2.787**	**	**0.032**	**0.184**
Direct effect	0.066	0.040	1.645	n.s.	−0.013	0.146
Indirect effect (Standard)	**0.042(0.067)**	**0.019(0.028)**	**–**	**–**	**0.007(0.015)**	**0.081(0.125)**

β = standardized coefficient; S.E. = standard error; *t* = t-value; p = probability value; LLCI = lower-level confidence interval; ULCI = upper-level confidence interval. Significance levels: **p < 0.01; n.s. = non-significant. Bold values indicate statistically significant results at *p* < 0.05.

#### Moderating effect of green marketing

**Figure 2:**
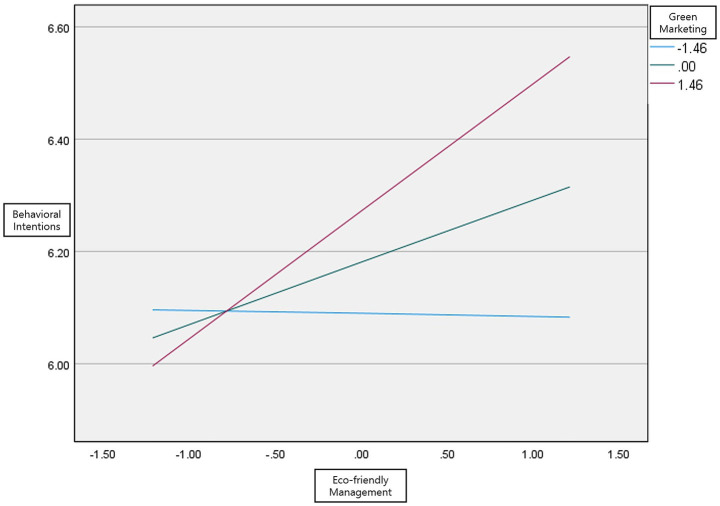
Moderating effect.

As shown in Table 8, the analysis of the moderating effect of green marketing indicates that eco-friendly management practices in golf clubs did have a direct effect on golfers’ behavioral intention (*β* = 0.080, *t* = 3.388, *p* < 0.01). According to the analysis of the influence of eco-friendly management on behavioral intention, two out of three (M-1SD, M, M + 1SD) intervals for green marketing—M (*β* = 0.111, *t* = 2.258, *p* < 0.05) and M + 1SD (*β* = 0.227, *t* = 3.460, *p* < 0.05)—did not include 0 between LLCI and ULCI, indicating statistical significance. This shows that the group with high green marketing awareness perceives eco-friendly management and behavioral intentions more positively than the group with low awareness, thereby resulting in a more positive perception of the golf club. In contrast, the low-awareness group does not view golf clubs positively through eco-friendly management. Therefore, the moderating effect of green marketing on the relationship between eco-friendly management and eco-friendly behavioral intention was confirmed (Figure 2).

**Table 8:** Analysis results of the moderating effect of green marketing.

Category		*β*	S.E.	*t*	*p*	LLCI	ULCI
Covariates	Gender	−0.020	0.109	−0.186	n.s.	−0.234	0.194
	Age	**0.163**	**0.059**	**2.769**	**	**0.047**	**0.279**
	Educational Background	0.012	0.066	0.179	n.s.	−0.118	0.142
	Experience	**0.337**	**0.140**	**2.412**	*	**0.062**	**0.612**
	Number of visits	−0.102	0.054	−1.903	n.s.	−0.207	0.004
Eco-friendly management		**0.111**	**0.049**	**2.258**	*	**0.014**	**0.207**
Green marketing		0.062	0.040	1.564	n.s.	−0.016	0.141
Eco-friendly marketing*Green marketing		**0.080**	**0.024**	**3.388**	**	**0.033**	**0.126**
*R*^2^ = 0.170, *F* = 6.233, *p* < 0.001							
−1 S.D.		−0.005	0.053	−0.102	n.s.	−0.111	0.100
Mean		**0.111**	**0.049**	**2.258**	*	**0.014**	**0.207**
+ 1S. D.		**0.227**	**0.066**	**3.460**	*	**0.098**	**0.356**

β = standardized coefficient; S.E. = standard error; *t* = t-value; p = probability value; LLCI = lower-level confidence interval; ULCI = upper-level confidence interval; S.D. = Standard Deviation. Significance levels: *p < 0.05, **p < 0.01; n.s. = non-significant. Bold values indicate statistically significant results at *p* < 0.05.

## Discussion

This study aims to examine the relationship between the eco-friendly management of golf clubs and behavioral intentions, focusing on the mediating roles of image and perceived quality, as well as the moderating effect of green marketing. The findings show that, first, a green image fully mediates the relationship between eco-friendly management and behavioral intention. Second, perceived quality also fully mediates this relationship. Third, green marketing has a moderating effect on this relationship.

First, the results of this study found that the environmental management activities of golf courses do not have a significant impact on golfers’ behavioral intentions, leading to the rejection of the hypothesis. This finding contrasts with the importance of green management emphasized in previous studies, and several interpretations can be made.

If consumers (golfers) are not sufficiently aware of the green management activities of the company (golf course), it is likely that these activities will not influence their behavioral intentions. According to Wiśniewska (2025), when consumers recognize eco-friendly policies and form positive attitudes toward them, behavior change is more likely to occur. However, the environmental management activities of golf courses are generally reflected in operational practices (e.g., using eco-friendly fertilizers, water conservation policies, introducing electric carts), which may be elements that golfers find difficult to directly perceive. Furthermore, there are various factors in choosing a golf course, and green management may not be the primary consideration for golfers. Lee and Lee (2022) found that key factors in choosing a golf course include course quality, price, accessibility, and service, while environmental factors had relatively less influence. In other words, golfers may prioritize play experience and facility quality over environmental protection. Beyond the prioritization of play quality and facility service, several other theoretical explanations may account for the insignificant direct effect of eco-friendly management on golfers’ behavioral intention. For instance, the visibility and emotional salience of green practices are often low in the golf context (Hartmann and Apaolaza-Ibáñez, 2012), limiting their influence on intention formation. Additionally, consumers may not recognize or perceive value in sustainability efforts unless clearly communicated (Testa et al., 2015), leading to underutilization of green cues in decision-making. The persistence of the value-action gap (Carrington et al., 2014) further suggests that pro-environmental attitudes alone are insufficient to drive behavior unless accompanied by strong situational or affective triggers.

Moreover, rather than directly changing golfers’ behavioral intentions, green management activities may have an impact through intermediary variables such as attitude change among consumers who value environmental issues. If the environmental management activities of golf courses attempt to influence behavioral intentions directly without going through attitude formation, their effect may be minimal. Therefore, further exploration is needed to understand how green management activities can lead to behavioral intentions through golfers’ attitudes and social norms.

Second, the green image of eco-friendly golf clubs fully mediates the impact of eco-friendly management on behavioral intention. That is, while eco-friendly management practices in golf clubs do not directly influence golfers’ behavioral intentions, behavioral intention is significantly affected by the green image of the eco-friendly golf club. These align with the findings of previous studies by Trang et al. (2019) and Wang et al. (2019), which found that eco-friendly management has a positive effect on consumers’ behavioral intentions and that a company’s image has an important role in this relationship. It also aligns with the findings of Lita et al. (2014), who found that consumer perceptions of corporate image positively impact eco-friendly behavior. The findings are also supported by Martínez et al. (2017), who demonstrated that an eco-friendly image positively influences consumer loyalty and future behavioral intentions.

These findings are also echoed in service and hospitality research. For instance, Genç and Zengin (2025) demonstrated that in eco-certified hotels, a strong green image significantly increased guests’ revisit intentions through trust and affective commitment. Likewise, Scro (2024) demonstrated that consumers are more likely to develop robust behavioral intentions when organizational sustainability efforts are not only emotionally salient but also resonate with the consumers’ self-identity and personal values. This alignment fosters a sense of psychological congruence, making eco-friendly behaviors feel more authentic, relevant, and intrinsically motivated. These examples highlight the importance of a positively perceived green image in various leisure and service contexts, supporting the current study’s findings.

However, this study found that there is a complete mediating effect through the green image of golf clubs. These findings can be explained on the basis of the theories of emotional connection and cognitive evaluation (Hui et al., 2021). By using eco-friendly golf clubs, golfers may feel a sense of satisfaction in contributing to environmental protection. Through the eco-friendly image, there is a potential to strengthen an emotional connection with the golf club. Moreover, the image of an eco-friendly golf club increases golfers’ trust in the golf club’s social responsibilities, playing a crucial role in driving behavioral intention.

The eco-friendly management of golf clubs, in terms of social responsibilities, promotes the awareness in golfers that the golf clubs are pioneering environmental improvement and positively contributing to enhancing the quality of life of each golfer (Coddington, 1993; Peattie, 1992). This solidifies their image as an eco-friendly golf club and positively impacts the stable growth of golf clubs.

Third, the perceived quality of eco-friendly golf clubs also fully mediates the relationship between eco-friendly management and behavioral intention. That is, while eco-friendly management practices in golf clubs do not directly influence golfers’ behavioral intentions, behavioral intention is significantly affected by the green image of the eco-friendly golf club. Previous studies are in line with these findings. Hashish et al. (2022) stated that the perceived eco-friendly quality of hotels has a direct impact on customers’ eco-friendly behaviors. Yusof et al. (2017) stated that eco-friendly efforts of hotels enhance customer satisfaction and loyalty; thus hotels must participate in sustainable environment preservation.

A key finding of this study is that the perceived quality of eco-friendly golf clubs completely mediates behavioral intentions. This suggests that even if eco-friendly management does not directly drive behavioral intention, perceived quality can play a decisive role in influencing golfers’ behavior. Perceived quality is related to the quality expected by customers before utilizing a service, and service can be enhanced through the eco-friendly management of golf clubs. For instance, golfers are more likely to expect a pleasant environment, clean facilities, and a harmonious experience with nature at golf clubs that practice eco-friendly management. Such expectations create a positive perception of the golf clubs, leading to behavioral intentions.

Fourth, green marketing by golf clubs moderates the relationship between eco-friendly management and behavioral intentions. When the level of green marketing (moderating variable) is low, eco-friendly management has little to no impact, or may have a negative effect, on behavioral intention. This suggests the possibility that consumers do not fully feel the effect of eco-friendly management, or management activities do not align with consumers’ personal values. Conversely, when the level of green marketing is high, the relationship between eco-friendly management and behavioral intentions strengthens, bringing about positive outcomes. This shows that consumers recognize the value of eco-friendly management and that green marketing plays an important role in enhancing behavioral intentions. These align with the findings of Puccinelli et al. (2009) that corporate eco-friendly practices often fail to translate into actual purchasing behaviors. However, Skackauskiene and Vilkaite-Vaitone (2022) argued that, in this relationship, a company’s green marketing strategies are essential factors that can regulate customer behavior. Furthermore, Ch et al. (2021) found that companies implementing eco-friendly strategies while ensuring robust green marketing achieve greater customer satisfaction. This is consistent with recent studies in the sports tourism sector. For instance, Huang and Chiu (2024) showed that green promotional campaigns during marathons significantly boosted runners’ loyalty and sustainable choices. And Chen (2025) identified green message framing as a key moderator between environmental practices and revisit intentions.

Therefore, while eco-friendly management of golf clubs has the potential to impact behavioral intentions, green marketing is essential to implement it. Green marketing plays an important role in consumers’ perceiving eco-friendly management activities as experiences connected to themselves. Marketing efforts should go beyond mere information provision; they must convince the consumers that their eco-friendly activities are connected to the values of each of the consumers.

This study has certain limitations. First, since the sample in this research was relatively small, the statistical power was insufficient. Therefore, further studies with a larger sample are necessary to confirm the findings. Second, there were regional constraints of golf courses in Korea and ambiguity surrounding the concept of eco-friendly golf courses. These factors restricted the scope of the study and reduced the generalizability of the results. Future research should address these limitations by establishing clearer criteria for eco-friendliness and securing a sample that reflects diverse regional characteristics.

## Practical implication

To effectively practice eco-friendly management at golf clubs, eco-friendly activities must be fully promoted, and strategies are needed to build a positive image. For instance, golf clubs may transparently disclose their eco-friendly policies and achievements or strengthen the emotional bonds with golfers by launching environmental protection campaigns in collaboration with local communities. Also, obtaining eco-friendly certifications or promoting green branding activities can foster trust among golfers and reinforce the golf clubs’ eco-friendly image.

Hence, for eco-friendly management of golf clubs to successfully drive behavioral intentions, they must strategically manage golfers’ perceived quality expectations. For this, the following practical strategies are proposed. First, experience-based marketing strategies must be used for the eco-friendly management of golf clubs to meet the expectations of golfers. For example, a special experience program in the eco-friendly golf club (e.g., ecological tours, environmental education) could be provided to enhance the golfers’ expectations. Second, eco-friendly management activities and outcomes may be promoted to solidify golfers’ expectations. For instance, the golf club’s recycling systems, energy-saving initiatives, and collaborations with the local community can be showcased using visual materials or on-site informational displays. Third, credibility can be strengthened by systematically certifying or disclosing the golf clubs’ eco-friendly operations of services. For instance, international eco-friendly certifications can be used or best practices of environmental protection can be shared to enhance golfers’ perceived quality expectations.

Therefore, golf clubs should implement eco-friendly course management, use sustainable materials, and offer opportunities for participation in eco-friendly programs. Stronger green marketing strategies are needed for consumers with a high interest in environmental issues (e.g., environmentally conscious golfers).

## Conclusion

This study aimed to investigate the relationship between the eco-friendly management of golf clubs and golfers’ behavioral intentions, focusing on the mediating roles of the image and perceived quality of eco-friendly golf clubs, as well as the moderating effect of green marketing. This study shows that golf clubs should not stop at simply implementing eco-friendly management practices but rather provide experience factors that satisfy customers’ experiences by promoting such practices effectively. Furthermore, it is important to strengthen the trust of the customers and enhance the clubs’ eco-friendly image and customers’ brand loyalty through green marketing.

### Recommendations for future studies

Future research should consider conducting comparative analyses across different types of golf facilities—such as full-scale golf clubs, practice ranges, and screen-golf venues—to explore how the nature of the service environment moderates perceptions of eco-friendly management. Such multi-group comparisons would offer deeper insights into the contextual sensitivity of green practices and may reveal distinct consumer response patterns that remain obscured in aggregate-level analyses.

As eco-friendly management and green marketing are broad concepts, future studies should define and distinguish the specific factors of eco-friendly management and green marketing. For instance, eco-friendly management factors can be distinguished as energy conservation, waste management, and ecosystem protection, and various green marketing strategies may be distinguished into customer-focused eco-friendly campaigns, sales of green products, and the provision of sustainable services. By doing so, each factor’s role and influence can be individually assessed, contributing to the enhancement of the paper’s empirical validity.

The moderating effect of green marketing may not be uniform across all consumer groups; rather, it is likely to vary depending on an individual’s level of environmental awareness. For instance, the effects of eco-friendly management and green marketing may differ between groups with high environmental awareness (High Environmental Awareness Group) and those with low environmental awareness (Low Environmental Awareness Group). Future research should consider conducting group-based analyses based on varying levels of environmental awareness.

The impact of eco-friendly management and green marketing on consumer behavior may also be influenced by cultural and regional factors. Therefore, future studies should explore cross-cultural comparisons (Cross-Cultural Study) or urban versus rural studies (Urban vs. Rural Study) to analyze how the effects of eco-friendly management differ based on environmental and cultural contexts.

Further studies may consider adopting a longitudinal research design to examine whether the moderating effects of green marketing continue over time. That is, it is important to analyze what long-term changes are brought about to consumers’ eco-friendly behaviors by the eco-friendly management of golf clubs and whether green marketing produces temporary effects or yields sustained influence. By doing so, whether green marketing drives positive behavioral intentions in the long run can be assessed.

Finally, it would be favorable to use diverse data collection methods, such as questionnaires, observational studies, in-depth interviews, and field investigations, to analyze in depth the mediating effects of golfers’ eco-friendly behavioral intentions and the green image of golf clubs. Employing a mixed methodology to collect both quantitative and qualitative data would enable a more comprehensive analysis of the research problem, further strengthening the reliability and robustness of findings.

## Data Availability

The raw data supporting the conclusions of this article will be made available by the authors, without undue reservation.
